# A modified particle swarm optimization algorithm for a vehicle scheduling problem with soft time windows

**DOI:** 10.1038/s41598-023-45543-z

**Published:** 2023-10-26

**Authors:** Jinwei Qiao, Shuzan Li, Ming Liu, Zhi Yang, Jun Chen, Pengbo Liu, Huiling Li, Chi Ma

**Affiliations:** 1 School of Mechanical and Automotive Engineering, Qilu University of Technology (Shandong Academy of Sciences), Jinan, 250353 People’s Republic of China; 2 Shandong Institute of Mechanical Design and Research, Jinan, 250353 People’s Republic of China; 3 Shandong Innovation and Development Research Institute, Jinan, 250353 People’s Republic of China; 4 Zaozhuang Xinjinshan Intelligent Equipment Co., Ltd, Zaozhuang, 277400 People’s Republic of China

**Keywords:** Mechanical engineering, Computational science, Statistics

## Abstract

This article constructed a vehicle scheduling problem (VSP) with soft time windows for a certain ore company. VSP is a typical NP-hard problem whose optimal solution can not be obtained in polynomial time, and the basic particle swarm optimization(PSO) algorithm has the obvious shortcoming of premature convergence and stagnation by falling into local optima. Thus, a modified particle swarm optimization (MPSO) was proposed in this paper for the numerical calculation to overcome the characteristics of the optimization problem such as: multiple constraints and NP-hard. The algorithm introduced the “elite reverse” strategy into population initialization, proposed an improved adaptive strategy by combining the subtraction function and “ladder strategy” to adjust inertia weight, and added a “jump out” mechanism to escape local optimal. Thus, the proposed algorithm can realize an accurate and rapid solution of the algorithm’s global optimization. Finally, this article made typical benchmark functions experiment and vehicle scheduling simulation to verify the algorithm performance. The experimental results of typical benchmark functions proved that the search accuracy and performance of the MPSO algorithm are superior to other algorithms: the basic PSO, the improved particle swarm optimization (IPSO), and the chaotic PSO (CPSO). Besides, the MPSO algorithm can improve an ore company’s profit by 48.5–71.8% compared with the basic PSO in the vehicle scheduling simulation.

## Introduction

The vehicle scheduling problem (VSP) is one of the most important scheduling problems in public transportation systems^1^, such as flight departure and arrival^2^, airport ground service support^3^, and school bus route planning^4^. Meanwhile, vehicle transportation for the ore company plays a very important role in the Mining process, which is the initial and essential stage of metallurgical engineering^5^. The main task of vehicle transportation is transporting the materials (such as ore) from the mining area or factory to an unloading station or a storage yard by different vehicles. The cost of vehicle transportation accounts for about 35–45% of the total cost in open-pit mines production^6^. The ore company needs to make suitable transportation routes for each vehicle by solving VSP quickly and accurately.

In general, VSP is classified as Vehicle Routing Problem (VRP) or Load Haul Dump problem (LHD)^7^. And, VSP is difficult to solve, because it can be viewed as an NP-hard problem whose optimal solution can not be obtained in polynomial time. VSP of ore company is a typical dynamic problem with the dynamic information disturbance by an external environment. Thus, the traditional solution method is not applicable. Besides, the traditional mathematical modeling method often fails to find feasible solutions because of the complexity of the model and the limitation of computing power.

The generation of particle swarm optimization (PSO) comes from the thinking of researchers after observing the team behavior in the process of bird predation^8^. Once proposed, this algorithm has attracted the attention and research of many scholars. The PSO algorithms has been widely utilized to address complicated issues in application areas like as engineering, finance, and computer science. For example, Mukhopadhyay and Banerjee^9^ proposed chaotic multi-swarm particle swarm optimization algorithm to optimize the parameters of the autonomous chaotic laser system. Jena et al.^10^ combined the PSO algorithm with an improved Q-learning algorithm to solve load balancing problems in cloud computing environment. Mariangela^11^ proposed an artificial neural network (ANN) together with PSO algorithm to select the optimal process parameters for the Micro electrical discharge machining process. Hao Feng et al.^12^ proposed an improved PSO algorithm to obtain the best Proportional-Integral-Derivative (PID) controller coefficients by solving the trajectory control problem of the electro-hydraulic position servo system. Xing et al.^13^ proposed an improved PSO algorithm to develop the energy consumption optimization model of tramway operation for reducing the traction energy consumption of the tramway. Wenyi Du et al.^14^ proposed an improved particle swarm optimization (PSO) algorithm to model the orderly charging strategy for the new energy vehicles (EV). Olmez et al.^15^ proposed the particle swarm with visit table strategy (PS-VTS) meta-heuristic technique to improve the effectiveness of Electroencephalogram (EEG)-based human emotion identification.

Similar to other swarm intelligence algorithms, the basic PSO algorithm, which is a non-globally convergent optimization algorithm, has poor diversity in the later stages and is easily prone to stagnation during the iteration process^16^. In application situations, PSO algorithms often experience the shortcoming of premature convergence and stagnation by falling into local optima. Therefore, many researchers have proposed corresponding improvement strategies to enhance the optimization ability of the algorithm. For example, Yue et al.^17^ proposed a modified PSO algorithm with a circular topology and it can form stable niches and locate multiple potential optimal solutions when solving multimodal multi-objective optimization problems. Gao et al.^18^ proposed a star-structured particle swarm optimization algorithm with a uniform calculation method for solving multimodal multi-objective problems. It has a closeness of over 95% compared to real Pareto frontiers. Solomon et al.^19^ designed a collaborative multi-swarm PSO algorithm for distributed computing environments. Simulation results showed that the PSO algorithm has high parallelism and achieved a maximum of 37 times speedup. Duan et al.^20^ designed an improved particle swarm optimization (IPSO) algorithm with nonlinear attenuation law and varying inertia weights to improve the coupling accuracy in laser-fiber coupling. Sun et al.^21^ proposed an improved particle swarm optimization algorithm by combining Non-Gaussian random distribution to optimize the design of wind turbine blades. Liu et al.^22^ introduced the differential evolution (DE) algorithm into PSO and proposed a hybrid algorithm called PSO-DE. Peng et al.^23^ proposed the symbiotic particle swarm optimization (SPSO) algorithm by adopting a multi population strategy.

In recent years, some researchers applied the PSO algorithms in the VSP fields. For instance, Rui et al.^24^ constructed an appropriate mathematical model for the typical vehicle-scheduling problem and proposed an improved immune particle swarm optimization with adaptive search(AS-ICPSO) strategy. Experimental results show that the proposed strategy can handle vehicle scheduling problem excellently. Hannan et al.^25^ proposed a modified particle swarm optimization (PSO) algorithm to solve a capacitated vehicle-routing problem. Sun et al.^26^ proposed a hybrid cooperative co-evolution algorithm (hccEA), in which a modified PSO is embedded into the cooperative co-evolution framework, to solve the vehicle scheduling problem with uncertain processing time. Xu et al.^27^ proposed a hybrid genetic algorithm and particle swarm optimization (PSO) for vehicle routing problem with time window, which decoded the path by particle real number coding method. It can avoid falling into local optimum.

In general, the basic PSO has been improved and developed by many researchers to date with many examples, and the improved methods can be classified into four categories: adjusting the distribution of algorithm parameters; changing the updating formula of the particle swarm position; modifying the initialization process of the swarm; combining with other intelligent algorithms. To improve the overall performance of the particle swarm algorithm, a modified particle swarm optimization (MPSO) is proposed for solving the multiple constraints and NP-hard vehicle scheduling problem. The MPSO algorithm is implemented under the cooperation of the following hybrid strategies: modifying the initialization process by the “elite reverse” strategy, changing the updating formula with an improved adaptive strategy, and adding the local optimal “jump out mechanism”. Compared with the other PSO algorithms, MPSO can avoid the resource wastes caused by population degradation and has good convergence accuracy and global search performance, especially when dealing with complex problems. This paper is presented as follows: “Formulation of VSP” presents the formulation of the vehicle scheduling optimization problem for a certain ore comp. The detailed strategies for the improvement of MPSO are described in “Modified particle swarm optimization algorithm”. In the “Simulation and discussion”, the benchmark and VSP simulations are given to verify the validity of the algorithm. “Conclusions” is given for a summary of this paper.

## Formulation of VSP

### Definitions and Declarations

1. Define the collection $J=\left\{ 1,2,\ldots , n \right\}$ represents the arrival order of vehicles, and *n* is the total number of vehicles;
2. Define the collection $R=\left\{ 1,2,\ldots , m \right\}$ represents vehicle types, and *m* is the total number of vehicle types, for instance: 1 means heavy vehicle, 2 means medium vehicle, and 3 means light vehicle;
3. Define variables $$y_{ij}={\left\{ \begin{array}{ll} 1,&{} \text {vehicle } i \text { and vehicle } j\text { is adjacent, and vehicle } i \text { is in front}\\ 0,&{} \text {other}\\ \end{array}\right. }$$
4. Define $E_j, L_j$ as the earliest and latest arrival times of vehicle *j*, where $j=1,2,\ldots ,n$;
5. Define $x_j$ as the actual arrival time for the vehicle *j*, where $j=1,2,\ldots ,n$;
6. Define $T_j$ as the expected arrival time for the vehicle *j*, where $j=1,2,\ldots ,n$;
7. Define $s^{rk}_{ij}$ as the safety time interval between the vehicle *i* and the vehicle *j*, where the vehicle *i* is vehicle type *r*, the vehicle *j* is vehicle type *k*, $r,k \in R$, $i,j \in J$ and the vehicle *i* is in front.
8. Define $z_{ij}$ as whether the vehicle *i* whose type is *r* and the vehicle *j* whose type is *k* are adjacent, where $i,j \in J,$ and $r,k \in R$.
9. Define $$\gamma _{ik}={\left\{ \begin{array}{ll} 1,&{} \text {vehicle } i \text { is vehicle type } k, \text { where} i \in J, k \in R \\ 0,&{} \text {other}\\ \end{array}\right. }$$
10. Defined $g_j, h_j$ as the unit time cost of early arrival or late arrival of the vehicle *j*.
11. Defined $\alpha _j= \max \left( 0,T_j-x_j \right) , \beta _j=\max \left( 0,x_j-T_j \right)$ as earliness of arrival and tardiness of arrival of the vehicle *j*.

### Modeling of VSP

The vehicle scheduling problem (VSP) for the ore company can be described as: there are *n* vehicles that need to enter the ore company for loading within a certain period, and the vehicles have a corresponding soft time window: earliest arrival time and latest arrival time. Within this soft time window, the company must meet both the production and quality requirements of ore production, as well as the total number of vehicles entering the site, and finally choose an optimal time for each vehicle as the arrival time of the vehicle. This paper mainly studies the VSP problem in the terminal area of the ore company, which means all vehicles enter the company by pairing approach. To ensure the safety of the vehicles‘ loading process, a certain safety separation must be maintained between vehicles. Because different types of vehicles may take different amounts of time to assemble ore and spend different amounts of time entering and leaving the yard, the safety interval between two adjacent vehicles is also different. In the process of building the vehicle scheduling model, most of the parameters are measured in terms of time, so we convert the safe interval between vehicles into a time interval to ensure the accuracy of the model calculation, as shown in Table 1.

**Table 1:** Time interval matrix between different types of vehicles.

Vehicle type		Front		
		Light	Medium	Heavy
Behind	Light	$s_{ij}^{11}$	$s_{ij}^{12}$	$s_{ij}^{13}$
	Medium	$s_{ij}^{21}$	$s_{ij}^{22}$	$s_{ij}^{23}$
	Heavy	$s_{ij}^{31}$	$s_{ij}^{32}$	$s_{ij}^{33}$

To ensure the safety of the vehicles and meet the basic production requirements, a scheduling sequence should be searched and optimized. Finally, our goal is to assign an optimal arrival time for each vehicle such that the following objective function is minimized.

1 $$\begin{aligned}{} & {} \min {\,\,}J =\,\,\sum _{j=1}^n{ \left( g_j \alpha _j+h_j \beta _j \right) } \end{aligned}$$

2 $$\begin{aligned}{} & {} st. E_j\le x_j\le L_j,\forall j\in J; \end{aligned}$$

3 $$\begin{aligned}{} & {} x_j=\beta _j- \alpha _j+T_j,\forall j\in J; \end{aligned}$$

4 $$\begin{aligned}{} & {} x_j\ge x_i+z_{ij}s^{rk}_{ij}-y_{ij}\left( L_j +s^{rk}_{ij} - E_i\right) ,\,\,\forall j,i\in J; \forall r,k\in R; j\ne i; \end{aligned}$$

5 $$\begin{aligned}{} & {} y_{ij}+y_{ji}=1,\,\,\forall j,i\in J; j \ne i; \end{aligned}$$

6 $$\begin{aligned}{} & {} z_{ij} \ge \gamma _{ir}+ \gamma _{jk}-1, \forall j,i\in J; i\ne j; r,k \in R; \end{aligned}$$

7 $$\begin{aligned}{} & {} \sum _{i=1}^m{\gamma _{ir}=1},\forall i \in J, \forall r\in R; \end{aligned}$$

8 $$\begin{aligned}{} & {} y_{ij}, z_{ij}, \gamma _{ir} \in \left\{ 0,1 \right\} , \forall j,i\in J; i\ne j, r \in R. \end{aligned}$$

Here, Eq. (1) minimizes the total penalty of arriving deviations from the target arriving time; Eq. (2) indicates the soft time windows for each vehicle. Eq. (3) link the decision variables $x_j$ and parameters $T_j$ to decision variables $\alpha _j$ and $\beta _j$; Eq. (4) represents the safety interval constraint of continuous arrival of vehicles. Given a pair of vehicles, Eq. (5) ensure one lands before the other. Eq. (6) links the decision variables $z_{ij}$ and $\gamma _{ir}$ and ensure the vehicle *i* whose type is *r* and the vehicle *j* whose type is *k* are adjacent; Eq. (7) ensure the uniqueness constraint of the vehicle type: the vehicle *i* can only be one of vehicle type: a heavy, a medium, a light vehicle, etc. The constraints (8) ensure that decision variables $y_{ij}, z_{ij}, \gamma _{ir}$ only take binary values.

## Modified particle swarm optimization algorithm

The problem of vehicle scheduling is a typical NP-hard problem, which is both multi-constraint and time-sensitive. If *n* vehicles are arriving in the company and ranking, there will be *n*! ranking orders. Estimating the cost of each ranking would be computationally time-consuming. Due to the simple principle, fast convergence speed, and easy programming of the PSO algorithm, some scholars have applied it to solve the vehicle scheduling problem, such as^28,29^.

In basic PSO algorithm, $X_i=[x_{i1}, \cdots ,x_{ij},\cdots ,x_{ND} ]$ and $V_i=\left[ v_{i1},\cdots ,v_{ij},\cdots ,v_{ND} \right]$ denote the position and velocity for each particle. Here, $i= 1,2,\cdots ,N$; *N* is the size of particle swarm; and $j = 1, 2, \cdots , D$, *D* is the dimension of the solution space. Besides, two important parameters are $p_{ij}$ and $g_{j}$. The former represents the personal best of particle *i*. The latter denotes the global best position tracked by the entire swarm. Then, the velocity updating Eq. (9) and the position updating Eq. (10) are given to adjust the search direction of the population.

9 $$\begin{aligned}{} & {} v_{ij}\left( k+1 \right) =wv_{ij}\left( k \right) +r_1c_1\left( p_{ij}-x_{ij}\left( k \right) \right) +r_2c_2\left( g_j-x_{ij}\left( k \right) \right) \end{aligned}$$

10 $$\begin{aligned}{} & {} x_{ij} (k+1)=x_{ij} (k)+v_{ij} (k+1) \end{aligned}$$

where, *w* is the inertia weight; $$ r_1,r_2 \in [0,1] $$ are uniformly distributed random numbers; $$ c_1,c_2$$ are the non-negative learning factors; $$ k \in [1,G]$$ is the current iteration step and *G* represents the maximum iterations.

Because the particles of the standard PSO algorithm are easy to fall into the local optimal solution, a modified PSO algorithm is proposed to solve the proposed vehicle scheduling problem.

### The “elite reverse” learning strategy

In the basic PSO algorithm, the population is initialized by a pure random strategy. However, the optimization accuracy and convergence speed are often limited by the random strategy. In this paper, the “elite reverse” learning strategy^30^ is introduced for the initialization to accelerate the algorithm’s solution speed and maintain the algorithm’s population diversity well. The specific operation is shown as follows: firstly, the initial population position matrix of the particle swarm generated by the random strategy is used, so that the elite solution vector of a single particle is $X=[x_1, \cdots ,x_j, \cdots ,x_D]$. Secondly, the calculation formula (11) is applied to obtain the elite reverse solution:

11 $$\begin{aligned} x_{ij} (k^{*})=k_r\left( u_{ij}+l_{ij} \right) -x_{ij}(k ) \end{aligned}$$

where $$u_{ij}, l_{ij}$$ represent the maximum and minimum values in the dimension *j* and $$ x_{ij}(k^{*})$$ represents the new particle position; $$k_r$$ is a random value that belongs to the interval (0,1). Finally, the fitness functions of the elite solution and the elite reverse solution are ranked, and the top *n* high-quality solutions are selected to form a new population position matrix.

### The mutation strategy from Genetic algorithm

In the iteration process of the basic PSO algorithm, the overall diversity of particle swarms would be reduced. To overcome this difficulty, the mutation strategy in the genetic algorithm^31^ is introduced to increase the diversity of individual extreme values and reduce the probability of particle swarms falling into the local optimum. The core of this strategy is to screen particles after each iteration, and the selected particles are applied by the position mutation formula (12).

12 $$\begin{aligned} x_{ij}^{*}\left( k \right) =x_{ij}\left( k \right) -wv_{ij}\left( k \right) -w \left( g_{j}-p_{ij} \right) \end{aligned}$$

where $x^*$ denotes the position of the particle after mutation.

### The adaptive weighting strategy

Inertia weight *w* is directly related to the convergence speed. The larger inertia weight *w* makes the particle have a stronger global search ability, and the smaller *w* makes the particle have better local search ability^32^. To improve the flexibility of particle flight speed change, an improved strategy combining the decreasing function and the “ladder” method is proposed to adjust the weight value. In the traditional “ladder” method, a constant value was chosen for each “ladder” which may lose a certain degree of flexibility. This paper proposes a “three-level ladder” adaptive strategy, in which the subtraction function method is applied for each “ladder”, to realize adaptive changes in each stage. The details of the switching formula are shown:

13 $$\begin{aligned} w={\left\{ \begin{array}{ll} w_{s1}-\left( w_{s1}-w_{e1} \right) \sqrt{\frac{K_1}{G}},&{} Fit1\le f\left( g \right) \\ w_{s2}-\left( w_{s2}-w_{s2} \right) \sqrt{\frac{K_2}{G}},&{} Fit2<f\left( g \right) <Fit1\\ w_{s3}-\left( w_{s3}-w_{s3} \right) \sqrt{\frac{K_3}{G}},&{} f\left( g \right) \le Fit2\\ \end{array}\right. } \end{aligned}$$

where$$ [w_{si}, w_{ei} ],i=1,2,3 $$ is the range of inertia weight; *f*(*g*) is the fitness function value corresponding to the global optimal solution; *Fit*1 and *Fit*2 are the autonomous set values, they are not fixed and unchanging but are determined by a comprehensive balance of the complexity of the optimized problem, the required optimization accuracy, and the PSO algorithm structure; $$ k_{1}, k_{2}, k_{3}$$ are the current iteration. The values of $$ [w_{si},w_{ei} ]$$ need to be adjusted according to the condition of the objective function in different application contexts. They are selected to provide a balance between local and global exploration and thus ensure the optimal solution can be found with a small number of iterations^33^. Thus, in the early stage, the particle swarm optimization algorithm should have a larger *w* value, so that the particle has a strong global optimization ability. The value of *w* gradually decreases in the later stage of the algorithm, so that the algorithm has better local search ability and improves the accuracy of the solution.

### The local optimal “jump out” mechanism

To avoid the phenomenon that the PSO algorithm easily falls into local optimum during the search process, the “jump out” mechanism is added. The criterion of falling into the local optimum is determined as: when the slope value of the global optimal fitness function curve is less than the specified value $\varepsilon$ in consecutive *m* iterations, it can be regarded as falling into the local optimum. The basic idea of the “jump out” mechanism is to be close to the global worst position and away from the global optimal position. The specific calculation formula is given as follows:

14 $$\begin{aligned} x_{ij}\left( k \right) =x_{ij}\left( k \right) -r_1c_1\left( g_{j}-x_{ij}\left( k \right) \right) +r_2c_2 \left( bad-x_{ij}\left( k \right) \right) \end{aligned}$$

where *bad* represents the information of the global worst position.

### The details of the algorithm process

The pseudo-code of the MPSO algorithm is demonstrated in Table 2.

**Table 2:** The pseudo-code of the MPSO algorithm.

Algorithm 1: The pseudo-code of the MPSO algorithm	
1:	Initialize the basic parameters: $N,D, m,G, \varepsilon , \ldots$
2:	Generate an initial population with the “elite reverse” learning strategy
3:	**while** $k \le G$ **do**
4:	Evaluate the fitness $f_k\left( g \right)$ for each individual
5:	Initialize $p_{ij}$,$g_{j}$ and *bad* among population
6:	**if** $( k \ge m \& \& \frac{\left( f_k\left( g \right) -f_{\left( k-m \right) }\left( g \right) \right) }{m} \le \varepsilon )$ **then**
7:	Jump out the algorithm by equation (14):
8:	$x_{ij}\left( k \right) =x_{ij}\left( k \right) -r_1c_1\left( g_{j}-x_{ij}\left( k \right) \right) +r_2c_2 \left( bad-x_{ij}\left( k \right) \right)$
9:	**else**
10:	Screen particles for mutation operation by Eq. (12):
11:	$x_{ij}^{*}\left( k \right) =x_{ij}\left( k \right) -wv_{ij}\left( k \right) -w \left( g_{j}-p_{ij} \right)$
12:	Calculate the fitness values of the new particle, and Update $p_{ij}$,$g_{j}$ and *bad*
13:	Update inertia weight *w* by Eq. (13)
14:	**end if**
15:	Update the position and velocity by Eqs. (9) and (10)
16:	$k: = k + 1$
17:	**end while**

**Figure 1:**
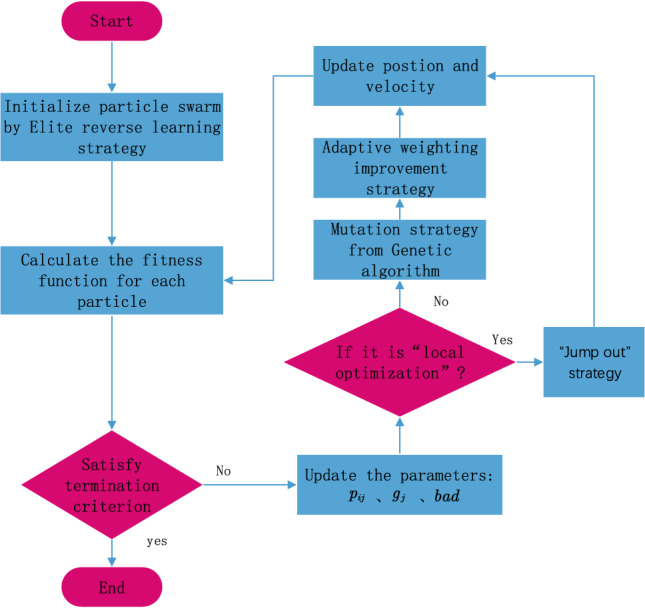
The overall flowchart of the MPSO algorithm.

The overall flowchart for the optimal placement of the MPSO is shown in Fig. 1. Besides, the specific steps and execution process are given as follows:

1. Initialize the basic parameters: particle swarm size, maximum number of iterations, inertia weight value, learning factor, and particle swarm dimension, etc.;
2. Generate initialized particle swarm positions according to the “elite reverse” learning strategy;
3. Calculate the fitness value of each particle according to the fitness function and determine whether the termination conditions are met, if yes go to step (8), otherwise go to step (4);
4. Update the parameters: $p_{ij}$, $g_{j}$, *bad* and determine whether to fall into the local optimum according to the criterion, if yes go to step (7), otherwise, go to step (5);
5. Screen particles for mutation operation and calculate the inertia weight value by Eq. (13);
6. Update the velocity and position of particles according to Eqs. (9) and (10) and jump to step (3);
7. Execute the position “jump out” strategy by Eq. (12) and jump to step (6);
8. End.

### Time complexity analysis

The time complexity of an algorithm is an important aspect to consider^34,35^. The computational complexity of the PSO algorithm is difficult to calculate precisely. It is mainly composed of the swarm size, the maximum number of iterations, and the complexity of the problem to be solved^36^.

According to Algorithm 1, the proposed MPSO algorithm can be divided into two main phases: first, the “elite reverse” learning strategy is used for the initialization of particles and velocity. The elites are first determined (Line 2), time complexity of this step is of order $$O(D^2)$$. Second, updating of particle position, and velocity and evaluating of fitness solution. The main loop of MPSO is executed for *G* iterations. Here, *N* dimensions are mutated per particle in the step (Lines 10), in which calculating the mutation probability per particle is of order*O*(1). Thus, the time complexity of the mutation operator is $$O(N*D)$$. The step (Lines 12) updates the $$p_{ij}$$ and the $$g_{j}$$, which is of order *O*(*D*). The step (Lines 13) of updating the parameters *w* is of order *O*(1). The velocity and position vectors of particles are updated in the step (Line 15). Based on Eq. (9), the time required for velocity updating per particle is of order $$O(D*N)$$. Furthermore, based on Eq. (10), the time complexity of position updating per particle is of order *O*(*D*). Thus, updating the velocity and position vectors of all particles is of order $$O(D*N)$$. The dominant step in each iteration is the mutation operator and the velocity updating of the swarm, which are with the same time complexity $$O(D*N)$$. The time complexity of other steps is relatively small and can be ignored compared to the above processes. Therefore, the total time complexity of the main loop of MPSO is of order $$O(N*G*D)$$.

## Simulations and discussion

To verify the effectiveness of the proposed MPSO algorithm, a benchmark function verification experiment and an ore vehicle scheduling optimization simulation are designed. Here, MPSO is compared and analyzed with other improved particle swarm optimization algorithms (PSO^37^, IPSO^38^, CPSO^39^). All simulations are implemented on a computer with Intel i5-5800H GPU, 1.80 GHz, and 16GB RAM. The codes are programmed by MATLAB R2018b.

### Validation of MPSO by benchmark test functions

**Table 3:** Description of unimodal and multimodal benchmark functions.

	Func	Name	Function’s expressions	Search range	$f_{min}$	D
Unimodal functions	F1	Sphere Problem	$f_1=\sum _{i=1}^n{ x_i ^2 }$	$[-100,100]^n$	0	50
	F2	Schwefel’s Problem 2.22	$f_2=\sum _{i=1}^n{\left| x_i \right| }+\prod _{i=1}^n{\left| x_i \right| }$	$[-10,10]^n$	0	50
	F3	Schwefel’s Problem 1.2	$f_3=\sum _{i=1}^n{ \left( \sum _{j=1}^i{ x_j }\right) ^2}$	$[-100,100]^n$	0	50
	F4	Schwefel’s Problem 2.21	$f_4=\max \left\{ \left| x_i \right| ,1\le i\le n \right\}$	$[-100,100]^n$	0	50
	F5	Rosenbrock ’s Problem	$f_{5}=\sum _{i=1}^{n-1}\biggl [100\biggl (x_{i+1}-x_{i}^{2}\biggr )^{2}+\biggl (x_{i}-1\biggr )^{2}\biggr ]$	$[-30,30]^n$	0	50
	F6	Step Problem	$f_{6}=\sum _{i=1}^{n}{\big (}{\big [}x_{i}+0.5{\big ]}{\big )}^{2}$	$[-100,100]^n$	0	50
	F7	Quartic Noise	$f_3=\sum _{i=1}^n{ix_{i}^{4}}+random\left[ 0.1 \right)$	$[-1.28,1.28]^n$	0	50
Multimodal functions	F8	Rastrigin	$f_8=\sum _{i=1}^n{\left[ x_{i}^{2}-10\cos \left( 2\pi x_i \right) +10 \right] }$	$[-5.12,5.12]^n$	0	50
	F9	Ackley	$f_{9}=20+e-20\exp \left( -0.2\sqrt{\frac{1}{n}\sum _{i= 1}^{n}x_i^2}\right) -\exp \left( \frac{1}{n}\sum _{i=1}^{n}\cos \left( 2\pi x_i\right) \right)$	$[-32,32]^n$	0	50
	F10	Griewank	$f_{10}={\frac{1}{4000}}\sum _{i=1}^{n}x_{i}^{2}-\prod _{i=1}^{n}\cos \left( {\frac{x_{i}}{\sqrt{i}}}\right) +1$	$[-600,600]^n$	0	50
	F11	Penalized’s Function	$f_{11}= \frac{\pi }{n}\bigg \{10{\text {sin}}\big (\pi x_{i}\big )+\sum _{i=1}^{n-}\big (x_{i}-1\big )^{2}\bigg [1+10{\text {sin}}^{2}\big (\pi x_{i+i}\big )\bigg ]+\big (x_{n}-1\big )^{2}\bigg \} +\sum _{i=1}^{n}u\big (x_{i},10,100,4\big )$	$[-50,50]$	0	50
	F12	Penalized’s Function	$f_{12}\left( x \right) = \frac{1}{10} \sin ^2\left( 3\pi x_1 \right) + \frac{1}{10} \sum _{k=1}^n{\left( x_k-1 \right) }^2\left[ 1+\sin ^2\left( 3\pi x_l+1 \right) \right] + \sum _{i=1}^n{u}\left( x_i,5,100,4 \right) + \frac{1}{10} \left( x_n-1 \right) ^2\left[ 1+\sin ^2\left( 2\pi x_n \right) \right]$	$[-50,50]$	0	50

There are two performance indicators to judge the optimization ability of intelligent algorithms: local development ability and global exploration ability. Thus, this paper selects two types of classical benchmark functions^40,41^, including seven unimodal (UM) functions ($$f_1,\ldots ,f_7$$ ) and five multimodal (MM) functions ($$f_8, \ldots , f_{12}$$ ). The names of each test function, mathematical formulation, and the global optimal solution are shown in Table 3. To ensure the fairness of the algorithm comparison, all parameters are concerning the original parameters in the relevant algorithm literature^37–39^. Some parameters of the proposed MPSO are listed as: $$[w_{s1}, w_{e1}]=[0.9,0.4] $$^32^, $$[w_{s2}, w_{e2}]= [0.65,0] $$^42^, $$ [w_{s3},w_{e3}]= [0.55,0.05] $$; $$V_{max}=0.1, V_{min}=-0.1 $$; $$ c_1 =2.5, c_2=1.5 $$, $$Fit1=10^6, Fit2=10^4$$ . The parameters related to jump-out local optimal are $$ s= 270 $$ and $$ \varepsilon = 0.001 $$. The relevant parameters for the PSO algorithms are shown in Table 4. All algorithms are repeated 30 times, the population size is 50, and the total number of iterations is 8000.

**Table 4:** Parameters of other PSO algorithms.

Symbol	Name	Size
N	Particle swarm size	125
D	Particle Swarm Dimension	50
G	Maximum number of iterations	8000
$w_s$	Initial value of inertia weights	0.8
$w_e$	Final value of inertia weights	0.05
$c_1$	Acceleration coefficient 1	2.5
$c_2$	Acceleration coefficient 2	1.5
$V_{max}$	Value of maximum particle’s velocity	0.1
$V_{min}$	Value of minimum particle’s velocity	−0.1

**Figure 2:**
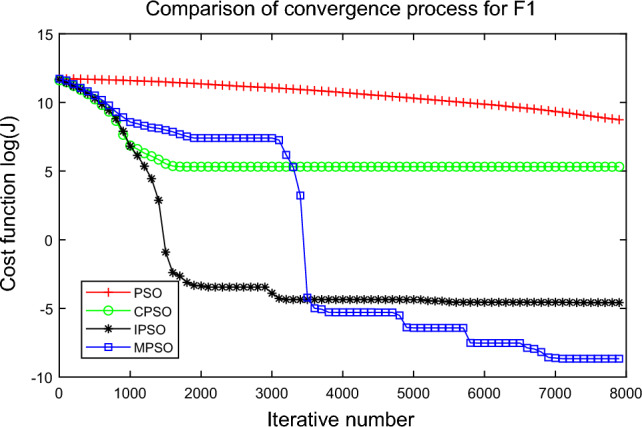
Comparison of performances of function F1 by four PSO algorithms.

**Figure 3:**
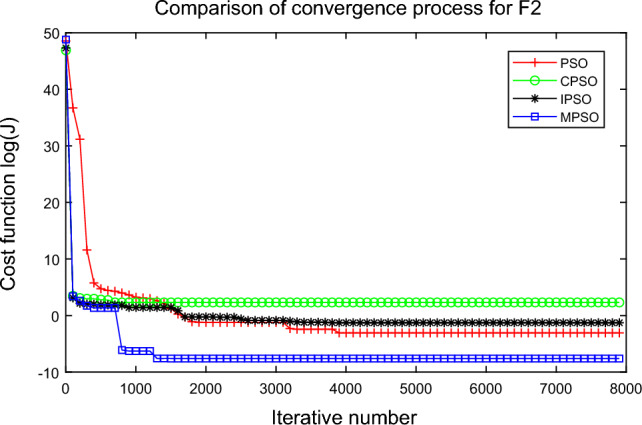
Comparison of performances of function F2 by four PSO algorithms.

**Figure 4:**
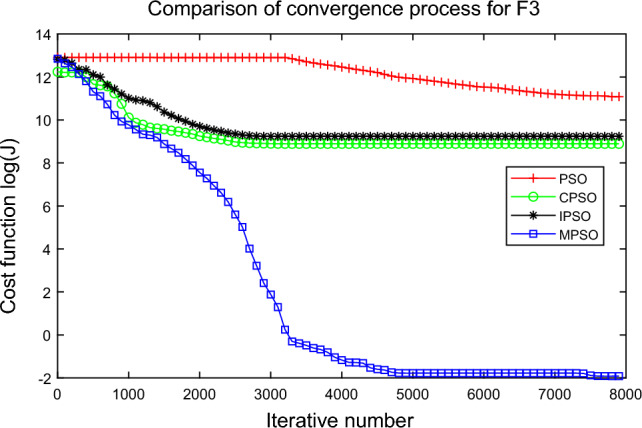
Comparison of performances of function F3 by four PSO algorithms.

**Figure 5:**
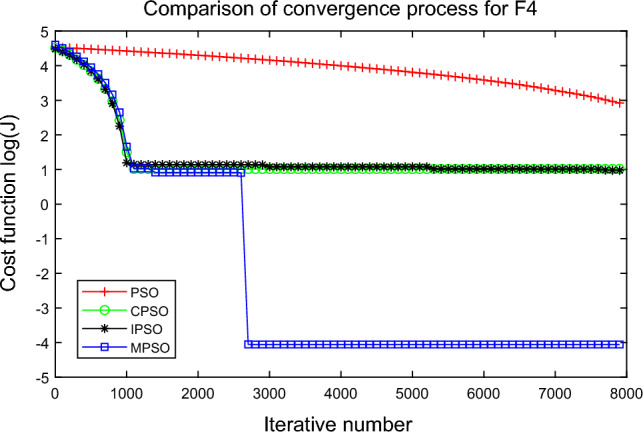
Comparison of performances of function F4 by four PSO algorithms.

**Figure 6:**
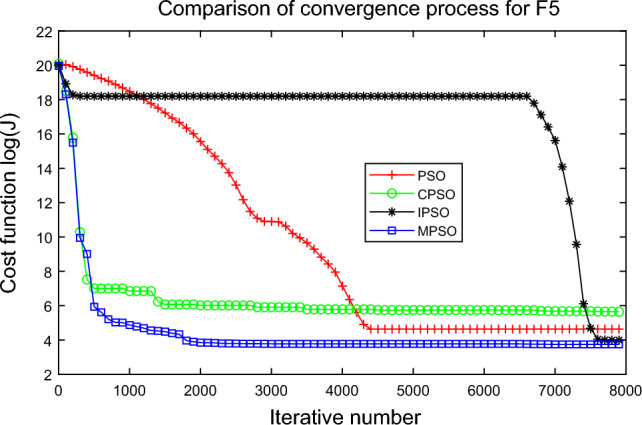
Comparison of performances of function F5 by four PSO algorithms.

**Figure 7:**
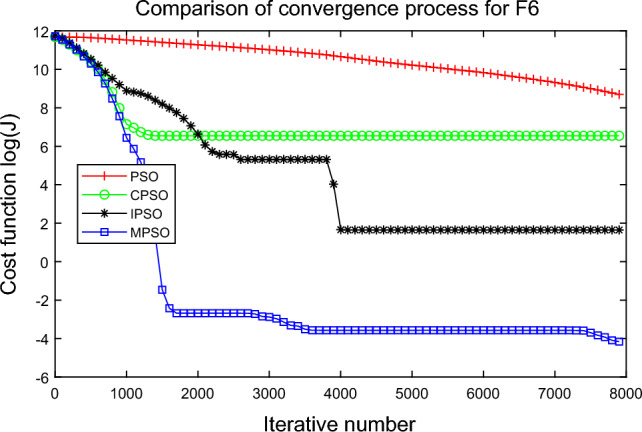
Comparison of performances of function F6 by four PSO algorithms.

**Figure 8:**
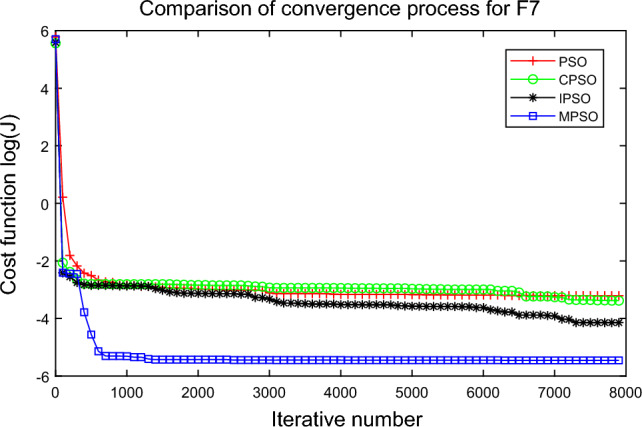
Comparison of performances of function F7 by four PSO algorithms.

**Figure 9:**
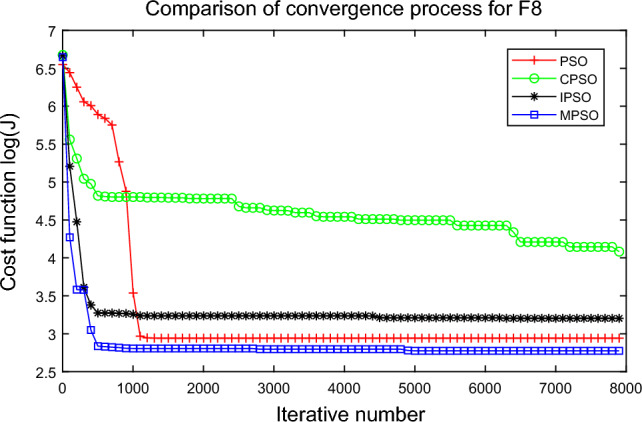
Comparison of performances of function F8 by four PSO algorithms.

**Figure 10:**
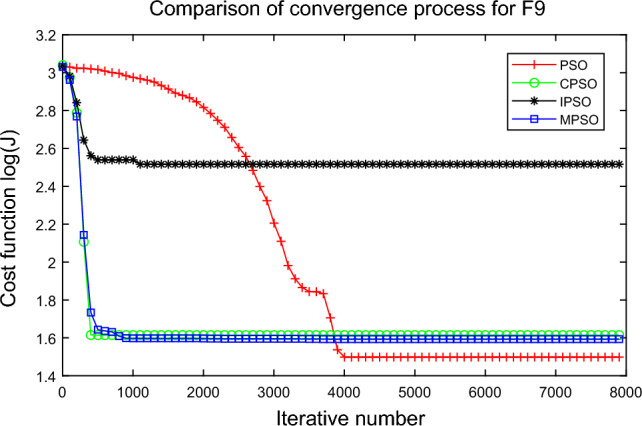
Comparison of performances of function F9 by four PSO algorithms.

**Figure 11:**
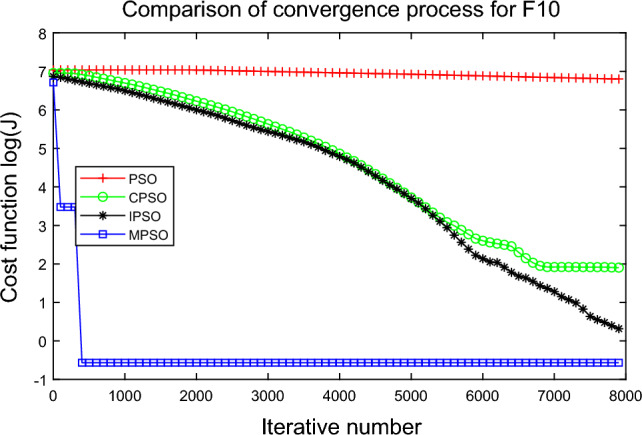
Comparison of performances of function F10 by four PSO algorithms. .

**Figure 12:**
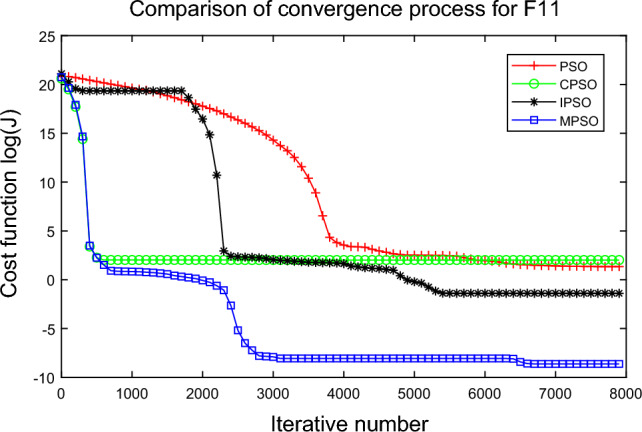
Comparison of performances of function F11 by four PSO algorithms.

**Figure 13:**
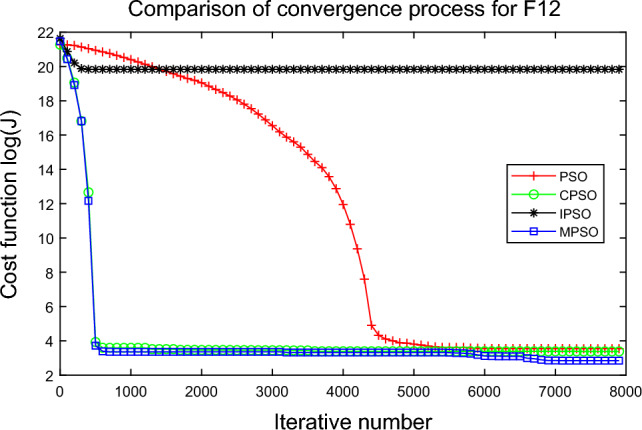
Comparison of performances of function F12 by four PSO algorithms.

In this experiment, the maximum value, the median value, the minimum value, the mean value and the standard deviation (SD) are used as the performance indicators to judge the optimization ability of the algorithm. The simulation results are shown in Table 5 and Figs. 2, 3, 4, 5, 6, 7, 8, 9, 10, 11, 12 and 13. The best value in Table 5 are shown in bold. The standard deviation reflects the stability of the algorithm, and the MPSO algorithm has obvious advantages for most of the functions F1–F12. Considering the UM benchmark functions, the results of (F1–F7) by MPSO perform better than other selected algorithms. For the MM functions (F8–F12), the best Mean values are also obtained by the MPSO algorithm. Based on the median and mean values of benchmark functions (F1–F12) in Table 5, high-quality solutions can be obtained by the MPSO algorithm.

**Table 5:** Results of benchmark functions.

Func	Alg	Cost function value				
		Max	Median	Mean	Min	SD
F1	PSO	7.31E+03	5.74E+03	5.81E+03	4.18E+03	6.44E+02
	CPSO	9.61E+02	3.09E+02	3.64E+02	1.24E+01	2.35E+02
	IPSO	1.00E+04	1.03E−02	7.00E+03	5.23E−03	4.66E+03
	MPSO	**3.66E**−**02**	**1.10E**−**02**	**1.18E**−**02**	**2.60E**−**03**	**7.89E**−**03**
F2	PSO	2.65E-01	7.22E-02	8.34E-02	3.01E-02	5.32E-02
	CPSO	3.35E+01	1.52E+01	1.60E+01	4.68E+00	6.51E+00
	IPSO	9.27E+00	1.46E−01	1.25E+00	1.61E−02	2.26E+00
	MPSO	**6.41E**−**02**	**8.65E**−**10**	**6.98E**−**03**	**4.51E**−**11**	**1.26E**−**02**
F3	PSO	7.20E+04	4.82E+04	4.70E+04	2.38E+04	1.29E+04
	CPSO	2.62E+04	6.98E+03	8.71E+03	8.90E+02	7.78E+03
	IPSO	3.41E+04	7.83E+02	1.48E+04	9.96E+03	6.05E+03
	MPSO	**6.19E+03**	**1.19E**−**01**	**1.36E+03**	**4.05E**−**02**	**1.69E+03**
F4	PSO	1.93E+01	1.82E+01	1.82E+01	1.68E+01	6.42E−01
	CPSO	5.72E+00	4.32E+00	4.42E+00	2.76E+00	7.45E−01
	IPSO	3.19E+00	2.24E+00	2.07E+00	1.09E+00	5.00E−01
	MPSO	**2.48E+00**	**7.37E**−**07**	**8.37E**−**02**	**6.60E**−**121**	**4.53E**−**01**
F5	PSO	7.99E+07	4.78E+01	4.94E+07	4.72E+01	3.84E+07
	CPSO	7.01E+02	2.37E+02	2.42E+02	6.14E+01	1.24E+02
	IPSO	1.55E+02	4.90E+01	6.80E+01	**4.14E+01**	3.36E+01
	MPSO	**1.13E+02**	**4.30E+01**	**5.31E+01**	4.18E+01	**1.78E+01**
F6	PSO	7.29E+03	5.74E+03	5.84E+03	4.48E+03	5.69E+02
	CPSO	1.18E+03	3.64E+02	4.32E+02	5.68E+01	2.39E+02
	IPSO	1.01E+04	1.01E+04	6.39E+03	**2.02E**−**05**	4.95E+03
	MPSO	**3.24E**−**02**	**1.23E**−**02**	**1.43E**−**02**	4.43E−03	**6.21E**−**03**
F7	PSO	5.90E−02	3.30E−02	3.49E−02	1.29E−02	1.18E−02
	CPSO	6.99E−02	3.87E−02	3.64E−02	1.27E−02	1.39E−02
	IPSO	6.74E−02	2.26E−02	2.79E−02	6.73E−03	1.32E−02
	MPSO	**1.15E**−**02**	**7.74E**−**04**	**2.78E**−**03**	**4.97E**−**05**	**3.23E**−**03**
F8	PSO	5.31E+01	3.44E+01	3.59E+01	1.89E+01	9.88E+00
	CPSO	1.42E+02	6.60E+01	7.00E+01	3.74E+01	2.28E+01
	IPSO	**5.31E+01**	3.37E+01	3.23E+01	1.77E+01	**7.92E+00**
	MPSO	7.86E+01	**1.24E+01**	**2.66E+01**	**1.60E+01**	1.41E+01
F9	PSO	6.69E+00	5.39E+00	5.41E+00	4.11E+00	6.50E-01
	CPSO	6.90E+00	5.86E+00	5.88E+00	4.19E+00	6.22E−01
	IPSO	1.27E+01	1.23E+01	1.23E+01	1.16E+01	**2.25E**−**01**
	MPSO	**6.00E+00**	**4.02E+00**	**4.91E+00**	**3.51E+00**	6.20E−01
F10	PSO	9.07E+02	8.52E+02	8.47E+02	7.24E+02	4.24E+01
	CPSO	1.64E+01	9.88E+00	9.47E+00	5.06E+00	2.74E+00
	IPSO	1.14E+01	4.19E+00	4.68E+00	1.28E+00	2.49E+00
	MPSO	**1.07E+01**	**5.68E**−**01**	**8.98E**−**01**	**5.22E**−**02**	**1.88E+00**
F11	PSO	1.40E+01	3.44E+00	4.37E+00	1.20E+00	2.86E+00
	CPSO	7.53E+00	1.63E+00	1.96E+00	1.73E−01	1.67E+00
	IPSO	2.56E+08	2.53E+08	1.78E+08	2.49E−01	1.18E+08
	MPSO	**3.59E+00**	**1.22E**−**04**	**3.11E**−**01**	**2.71E**−**05**	**7.87E**−**01**
F12	PSO	5.86E+01	3.72E+01	3.81E+01	2.11E+01	9.96E+00
	CPSO	4.74E+01	3.49E+01	3.62E+01	2.45E+01	**6.17E+00**
	IPSO	4.10E+08	4.09E+08	3.00E+08	1.03E+01	1.84E+08
	MPSO	**4.51E+01**	**2.13E+01**	**2.95E+01**	**1.39E+01**	8.54E+00

The average fitness values of the optimal solution of each algorithm are plotted to compare the performance of each algorithm more clearly and intuitively, as shown in Figs. 2, 3, 4, 5, 6, 7, 8, 9, 10, 11, 12 and 13. Taking function F2 as an example, the changes in the adaptation value *log*(*J*) of the four algorithms in Fig. 3 are analyzed in detail. The CPSO algorithm declined rapidly in the first 200 generations, but could not jump out after falling into “local optimization”, resulting in the largest *log*(*J*) value of this algorithm and the worst convergence accuracy of the algorithm. The PSO algorithm can reach the optimal solution around 4000 generations, and the particles of the IPSO algorithm reach the optimal solution around 2500 generations. The particles fall into “optimization” around the 700th to 1200th generations in the MPSO algorithm, but the “jump-out” strategy of the algorithm increases the possibility of other searches in the direction of the optimal solution, and finally, the optimal solution is obtained around the 1300th generation. The results show that the search speed and search accuracy of the MPSO algorithm are improved and better than the other three algorithms.

Based on Figs. 2, 3, 4, 5, 6, 7 and 8, the convergence speed of the MPSO algorithm is significantly faster than that of other algorithms when the unimodal test functions are solved. Besides, the *log*(*J*) value of the MPSO algorithm is the lowest, which indicates that the optimization accuracy of the MPSO algorithm is higher than other algorithms. Figs. 9, 10 , 11, 12 and 13 show that when the MPSO algorithm is used to solve the multimodal test function, it quickly converges to a small optimal range after about 300 iterations, and its convergence speed is much greater than that of other algorithms, and the optimal solution value is significantly lower than that of other algorithms. Thus, the MPSO algorithm has a fast convergence speed and high global search capability for multimodal functions. Finally, the MPSO algorithm has better convergence than the other three PSO algorithms for solving different test functions.

**Figure 14:**
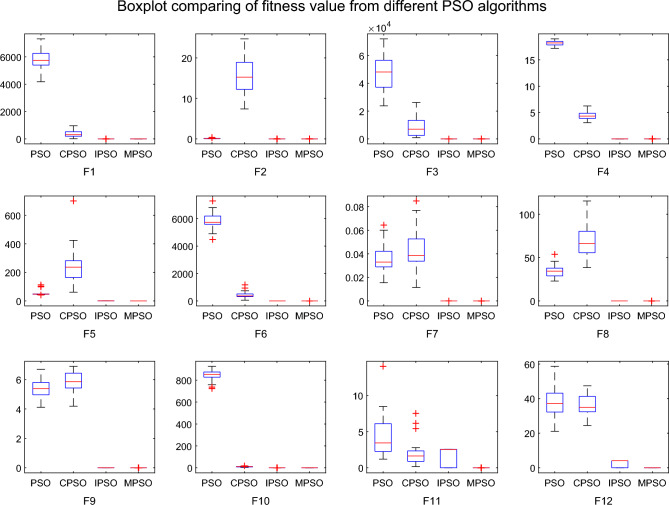
Boxplot comparing of cost function by four different PSO algorithms.

To evaluate the performance of different PSO algorithms, statistical tests should be conducted^43^. In general, the results of an optimization algorithm cannot be distributed normally. Due to the stochastic nature of the meta-heuristics, it is not enough to compare algorithms based on only the mean and standard deviation values^44,45^. When the optimization results cannot be assumed to obey the normal distribution, a non-parametric test for comparison is necessary to judge whether the results of the algorithms differ from each other in a statistically significant way. Thus, the Wilcoxon non-parametric statistical test^46^ is used by u to obtain a parameter called p-value to verify whether two sets of solutions are different to a statistically significant extent or not. Generally, it is considered that $p \le 0.5$ can be considered as a statistically significant superiority of the results. The p-values calculated in Wilcoxon’s rank-sum test comparing MPSO and other PSO algorithms are listed in Table 6 for all benchmark functions. The p-values in Table 6 additionally present the superiority of the MPSO because all of the p-values are much smaller than 0.05. Besides, Fig. 14 shows a set of box-plots of performance comparisons of all algorithms for the benchmark functions of F1 to F12. From Table 6 and Fig. 14, it is obvious that the MPSO has superior performance in terms of solving unimodal and multimodal functions

**Table 6:** Results of the p-value for the Wilcoxon rank-sum test on benchmark functions.

Algorithm	F1	F2	F3	F4	F5	F6
PSOvsMPSO	1.91E−07	3.26E−07	1.61E−06	9.71E−07	1.68E−07	4.79E−07
CPSOvsMPSO	2.97E−07	9.18E−07	6.57E−07	1.36E−06	1.15E−06	1.69E−06
IPSOvsMPSO	9.40E−05	4.61E−05	4.45E−06	1.82E−07	5.46E−04	1.04E−04

Algorithm	F7	F8	F9	F10	F11	F12
PSOvsMPSO	1.36E−06	7.25E−07	1.17E−06	8.42E−07	1.39E−06	1.47E−06
CPSOvsMPSO	1.56E−06	1.65E−06	1.51E−06	1.20E−06	1.61E−06	9.97E−07
IPSOvsMPSO	1.51E−06	8.33E−07	6.79E−07	1.00E−06	1.99E−05	5.51E−06

### Vehicle scheduling optimization simulation

A total of 10 ore vehicles (of which vehicles 1 and 2 are light vehicles, and vehicles 3–10 are medium-sized vehicles) are considered in this experiment. The earliest and latest arrival times of vehicles are shown in Table 7, the interval constraints of the arrival time of adjacent vehicles are given: $s_{ij}^{11}=3,s_{ij}^{12}=15,s_{ij}^{21}=15,s_{ij}^{22}=8$. The MPSO algorithm is compared with not only other PSO algorithms(PSO^37^, IPSO^38^, CPSO^39^) but also four state-of-the-art meta-heuristic methods(WOA^47^, IA^48^, DE^49^, ABC^50^) on the vehicle scheduling problem.

**Table 7:** The earliest and latest arrival times of each vehicle.

Vehicle	1	2	3	4	5	6	7	8	9	10
Time $l_j$	2:09	3:15	1:29	1:36	1:50	2:00	2:04	2:06	2:15	2:40
Time $u_j$	9:19	12:24	8:30	8:41	9:15	9:36	9:37	9:33	9:51	10:57

#### Comparison of MPSO and Other PSO Algorithms

**Figure 15:**
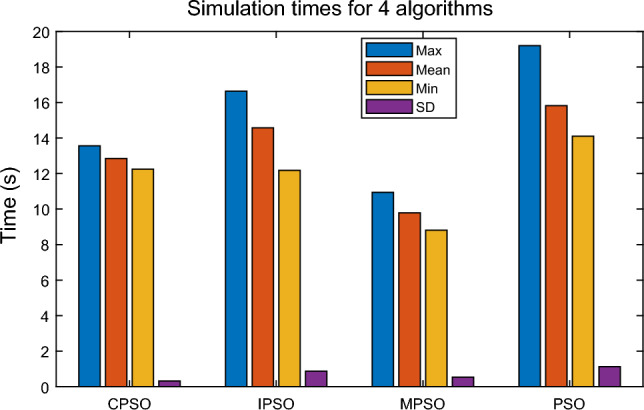
Comparison of simulation times for four PSO algorithms.

**Figure 16:**
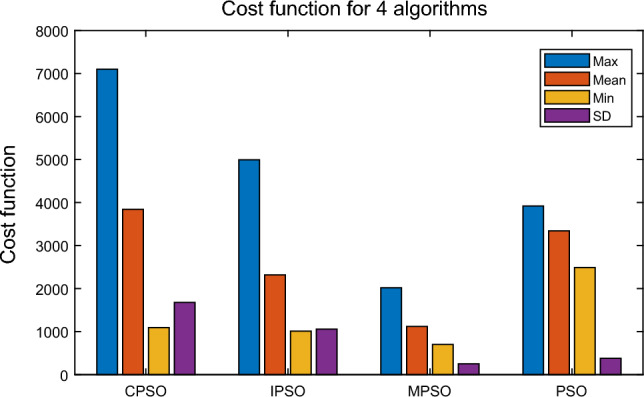
Comparison of cost function values for four PSO algorithms.

**Figure 17:**
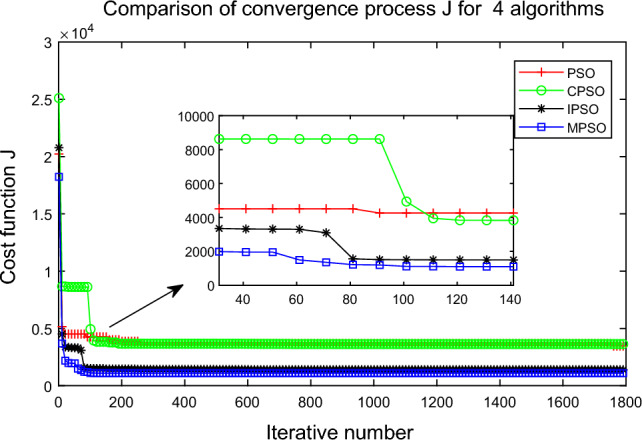
Comparison of convergence process for four PSO algorithms.

Due to the random initialization of the PSO algorithms, the algorithms (PSO^37^, IPSO^38^, CPSO^39^, and MPSO) are repeated 30 times, and the total number of iterations is 1800. Each algorithm is evaluated by the mean, maximum, minimum, and standard deviation. The simulation results are shown in Figs. 15, 16 and 17 and Table 8.

It can be seen from Fig. 15 and Table 8 that the simulation time of the MPSO algorithm is significantly better than that of PSO, CPSO, and IPSO algorithms. The maximum, minimum, and average values of the 30 operations of the MPSO algorithm are better than the other three algorithms, and the standard deviation of the calculation time is only less than the CPSO algorithm. The maximum value of simulation time by MPSO is 10.94 seconds, it can improve the algorithm’s computation time profit by 42.9% compared with the basic PSO. The minimum value of simulation time by MPSO is 8.82 seconds, the algorithm’s computation time is improved by 37.4% compared with the basic PSO. Thus, MPSO can improve the algorithm’s computation time profit by 37.4–42.9% compared with the basic PSO. The comparison results indicate that the convergence efficiency of the MPSO algorithm is high.

From Fig. 16 and Table 8, the final objective function value (*J*) of the MPSO algorithm is significantly lower than that of PSO, CPSO, and IPSO algorithms, and the standard deviation SD of the final objective function value of the MPSO algorithm has the best stability. The maximum value of objective function value (*J*) by MPSO is 2020.47, it can improve an ore company’s profit by 48.5% compared with the basic PSO. The minimum value of *J* by MPSO is 702.03, and the ore company’s profit is raised by 71.8% compared with the basic PSO. In summary, MPSO can improve an ore company’s profit by 48.5%-71.8% compared with the basic PSO. Thus, the MPSO algorithm can obtain the best optimization scheduling results, save resource consumption for enterprises, and effectively reduce the workload of vehicle scheduling.

**Table 8:** The calculation results of each algorithm for vehicle scheduling simulation.

Algorithm	Simulation time (s)				Cost function value			
	Max	Mean	Min	SD	Max	Mean	Min	SD
PSO	19.19	15.82	14.1	1.13	3921.01	3342.87	2488.7	377.73
CPSO	13.56	12.85	12.24	**0.32**	7101.63	3840.47	1090.32	1678.46
IPSO	16.64	14.57	12.18	0.87	4992.79	2316.66	1009.7	1055.47
MPSO	**10.94**	**9.79**	**8.82**	0.54	**2020.47**	**1118.26**	**702.03**	**250.4**

The best values are shown in bold.

In Fig. 17, the MPSO algorithm can converge well in the early stage, and its distribution proves that the MPSO algorithm can quickly escape the local optimum. They can also verify the effectiveness of avoiding “precociousness” by related proposed improvement strategies in the MPSO algorithm.

In general, MPSO outperforms other PSO algorithms on the VSP optimal problem. The reason for this behavior is likely that MPSO is able to choose the most suitable strategy for different search stages. The adaptive weighting strategy of dynamic weight is given to improve the global search speed. Besides, and the criterion of falling into the local optimum and a “jump out” strategy are interactive to overcome the “premature” problem.

#### Comparison of MPSO and other meta-heuristic algorithms

In order to determine the place of the proposed MPSO method, the proposed MPSO method is compared with 4 state-of-the-art meta-heuristic methods (WOA^47^, IA^48^, DE^49^, ABC^50^) on the vehicle scheduling problem. The parameters of these algorithms are listed in Table 9. Each algorithm was tested 30 times independently to reduce statistical errors.

**Table 9:** Parameters of other optimization algorithms.

Algorithms	Population	Maxi Iteration	Dim	Other
WOA	125	1800	10	$r_1,r_2 \in [0,1]$ are random numbers
IA	125	1800	10	$p_m=0.7, \alpha = \beta =1, \delta =0.2, ncl=10$
DE	125	1800	10	$$F0=0.4, CR=0.1 $$
ABC	125	1800	10	$\alpha =1$

The comparison of simulation time and final cost value between MPSO and other meta-heuristic methods is shown in Table 10, in which the mean, maximum, minimum, and standard difference of simulation results were recorded and shown. The best results are shown in bold type. As one can see in Table 10, by utilizing the proposed strategy based on the MPSO, the lowest final cost value is obtained. The simulation time of WOA is the lowest. By contrast, MPSO spends some computational cost to perform execution on the criterion of falling into the local optimum and the “jump out” strategy. However, the final cost function value of WOA is the highest, which means it is inherently unreliable by having traded speed for accuracy. Table 10 proves that MPSO can obtain the lowest cost function value and the simulation time is also lower than the other three meta-heuristic methods. By comprehensive comparison, the solution of the MPSO algorithm gives the best value.

**Figure 18:**
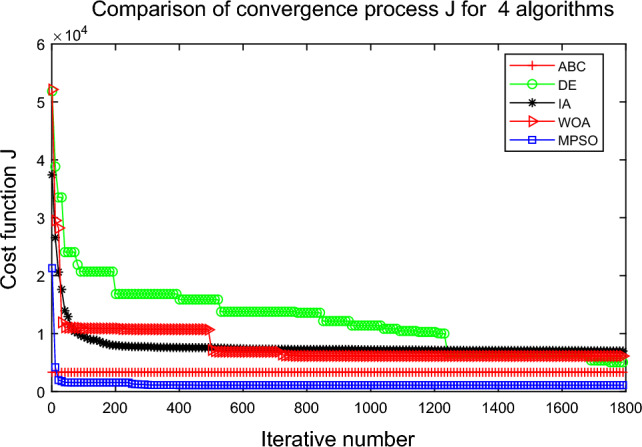
Comparison of MPSO with other optimization algorithms.

The convergence graph of each algorithm is shown in Fig. 18. In Fig. 18, the MPSO algorithm is more successful than all of the other optimization approaches, and the algorithm determines the global optimal solution after approximately 30 generations.

**Table 10:** The calculation results of each algorithm for vehicle scheduling simulation.

Algorithm	Simulation time (s)				Cost function value			
	Max	Mean	Min	SD	Max	Mean	Min	SD
ABC	8.53	8.19	7.90	0.17	4776.15	3419.52	3322.62	368.77
DE	1.38	1.22	1.03	0.08	7328.57	4937.21	3409.88	1114.26
IA	8.25	7.43	7.07	0.24	17913.74	14794.28	9858.98	1956.00
WOA	**0.22**	**0.17**	**0.13**	**0.02**	22449.33	9674.24	2466.88	5120.64
MPSO	1.07	0.93	0.84	0.05	**1390.00**	**1096.59**	**702.08**	190.21

The best values are shown in bold.

## Conclusions

In this paper, the MPSO algorithm was proposed to solve a vehicle scheduling optimization problem with soft time window constraints for a certain ore company. The multiple swarm scheme, which combines the “elite reverse” strategy, an improved adaptive strategy, and the local optimal “jump out mechanism”, was introduced into the MPSO algorithm, The validity and feasibility of the MPSO were verified by 12 classical benchmark functions and an ore vehicle scheduling optimization simulation. The following conclusions are given.

- The benchmark results indicate that the MPSO algorithm has superior performance than other PSO algorithms (PSO, IPSO, CPSO).
- The MPSO algorithm can improve an ore company’s profit by 48.5%-71.8% compared with the basic PSO. It can obtain the best optimization scheduling results, save resource consumption for enterprises, and effectively reduce the workload of vehicle scheduling.

Consequently, the paper verifies the feasibility of the MPSO algorithm and the success of solving a vehicle scheduling optimization problem for a certain ore company and provides a theoretical basis for subsequent research. Next, the following three issues will be studied: Firstly, the tasks and load balancing should be considered during the modeling process. Secondly, the performance of the proposed MPSO strategy can be improved by introducing other intelligent algorithms, such as the differential evolution algorithm. Finally, the proposed algorithm will be applied in a real ore company environment.

## Data Availability

The data that support the findings of this study are available from [Zaozhuang Xinjinshan Intelligent Equipment Co., Ltd] but restrictions apply to the availability of these data, which were used under license for the current study, and so are not publicly available. Data are however available from the authors upon reasonable request and with permission of [Zaozhuang Xinjinshan Intelligent Equipment Co., Ltd].
